# Water temperature dynamics in a headwater forest stream: Contrasting climatic, anthropic and geological conditions create thermal mosaic of aquatic habitats

**DOI:** 10.1371/journal.pone.0281096

**Published:** 2023-02-15

**Authors:** Paul Bois, Jean-Nicolas Beisel, Alban Cairault, Nicolas Flipo, Corentin Leprince, Agnès Rivière

**Affiliations:** 1 CNRS, ENGEES, ICube UMR 7357, Université de Strasbourg, Strasbourg, France; 2 CNRS, LIVE UMR 7362, Université de Strasbourg, Strasbourg, France; 3 ENGEES, Strasbourg, France; 4 Parc Naturel Régional des Vosges du Nord, Château, F-La Petite Pierre, France; 5 Geosciences Department, Mines ParisTech, PSL University, Fontainebleau, France; University of Eldoret, KENYA

## Abstract

The thermal regime of streams is a relevant driver of their ecological functioning. As this regime is presently submitted to numerous alterations (among others, impoundments, and climate change), it seems important to study both their effects and potential recovery from the latter. Thus, we investigated the surface and hyporheic water temperature along a small headwater stream with contrasting environmental contexts: forest landscape, open grassland landscape without riparian vegetation, several artificial run-of-the-river impoundments and one discharge point of a by-pass impoundment. The main objectives were to study the influence of these contrasting contexts on surface and subsurface water temperature at a local scale. Contrasting contexts were supposed to create effects on both surface and hyporheic thermal regimes at a local scale. Differences of thermal regimes between surface and hyporheos were expected, as well as between geological contexts. Sensors located at multiple stations allowed monitoring of stream and hyporheos temperature along the stream, while comparison with adjacent reference stream allowed for surface water thermal regime benchmark. Impoundments and landscapes significantly influenced stream thermal regime at a local scale (impoundments created up to +3.7°C temperature increase in average). Their effect on hyporheos thermal regime was less marked than the ones generated by solar radiation or geological features. Hyporheos thermal regime varies from stream one by temperature dynamics delay (up to 18h) and decrease (up to -7°C between surface and hyporheos temperature in average). These coupled effects create a mosaic of thermal habitats, which could be used for river biodiversity preservation and restoration.

## Introduction

A disproportionally large fraction of the world’s total biodiversity is composed of ectotherm animals: their body temperature is variable and directly linked to that of the surrounding environment. Freshwater aquatic species constitute around 9.5% of the total number of animal species globally recognized, distributed on only 0.01% of the total Earth surface [1]. Even if an animal produces heat as a by-product of metabolic reactions, thermal energy accumulates in the aquatic environment with moderated temperature increase thanks to the large thermal capacity of water. The metabolism (e.g., respiration, digestion, muscle activity, and photosynthesis) and the life cycle of ectotherms closely depend on the temperature of their habitat. A change of water temperature thus has a direct impact on the maintenance of freshwater species and on aquatic diversity of an ecosystem.

By nature, freshwater temperature greatly varies in space and time under the effects of major physical drivers. The major factor that determines freshwater temperature is the energy flux at the air/surface water interface through: (i) solar input or net short-wave radiation; (ii) net long-wave radiation; (iii) latent heat flux (evaporation); and (iv) sensible heat flux (resulting from temperature difference between the river and the atmosphere) [2, 3]. Modifications to the energy budget and/or the thermal inertia consequently alter the natural thermal regime of a stream i.e., the distribution of the magnitude of water temperature, the frequency with which a given temperature occurs, the time of the year when a certain temperature occurs, and the time during which a stream is above or below a given temperature [4, 5].

The stream-aquifer interface, defined as the mixing zone between groundwater and surface water (SWGW) (also called hyporheic zone), has also been identified as largely influencing stream heat budgets [5, 6], although its relative importance can be low compared to solar input [7]. Temperature profiles within this interface reflect the stream-aquifer hydraulic exchanges and the thermal gradient between the stream and aquifer. For many small instars of aquatic organisms, the SWGW interface is also a refuge from extreme water temperature conditions that occur in the surface stream [5, 8]. Gradients of nutrients, organic matter and physico-chemical processes occur in response to variations in discharge and porosity [9]. Hence, this active ecotone is of importance for the functioning and the biodiversity of rivers [10].

Water temperature can be seen as a measure of the thermal energy content in a stream, that depends on both energy fluxes and stream discharge [5]. Any process that influences energy fluxes to the channel or discharge in the channel will thus influence channel water temperature and can be considered a driver of stream temperature [5]. Hence, impoundments and riparian alterations modify thermal regimes of freshwater ecosystems through modifications of water energy fluxes [11–13]. This is mostly important in headwater streams with Strahler order of 1 or 2 [14], where thermal inertia is limited by its reduced discharge. Additionally, to date the effect of these run-of-the-river impoundments on the thermal regime of hyporheic flow has never been described.

Northeast of France is the country region where rivers have the highest number of barriers to flow through: at least 63 each 100 km of rivers in average (link to website, read online on 10/26/2021). Among them many are artificial run-of-the-river ponds that were constructed in the 1960’s and 1970’s by placing a weir to retain water upstream. This profoundly modified the natural flow of water and sediments and influenced the thermal regime: a pond receives a high quantity of short-wave radiation and water residence time inside the pond is much longer than on the pristine river. When they are shallow, they usually show no stratification, and act as heat accumulators along the stream.

In this study, we investigated surface and subsurface water temperature along a small headwater stream characterized by contrasting environmental contexts, due to 1) landscape features (alternating open grassland and forest), 2) hydrogeological features (varying geological types) and 3) stream management features (presence of run-of-the-river and by-pass impoundments along the stream). More specifically, we made three hypotheses related to these features:

- Landscape features may generate differential radiation energy inputs depending on the land cover, resulting in more thermal amplitude (warmer maximum temperature, colder minimum temperature) in open grassland landscape than in forest landscape
- Hydrogeological features may generate differential hyporheic energy and water exchanges due to varying thermal and hydraulic conductivities, and porosities, resulting in reduced magnitude and time shifts of hyporheic temperature in highly conductive sections and larger shifts in lowly conductive sections of the stream
- Stream management features may generate differential energy inputs in the stream due to slowed down water in run-of-the-river impoundments, with a well-known effect of warming/cooling depending on the outflow point location, and temperature alteration due to outflow of by-pass impoundments.

Based on these three hypotheses, we wish to explore two parallel questions:

1. What is the influence of these contrasting contexts on stream (i.e., surface water) temperature at a local scale?
2. What is the influence of these contrasting contexts on hyporheos (i.e., subsurface water) temperature at a local scale?

Put differently, the main goal here is to study to what extent these features lead to a mosaic of thermal habitats within the stream, both longitudinally and vertically.

## Material and methods

### Study site

**Fig 1:**
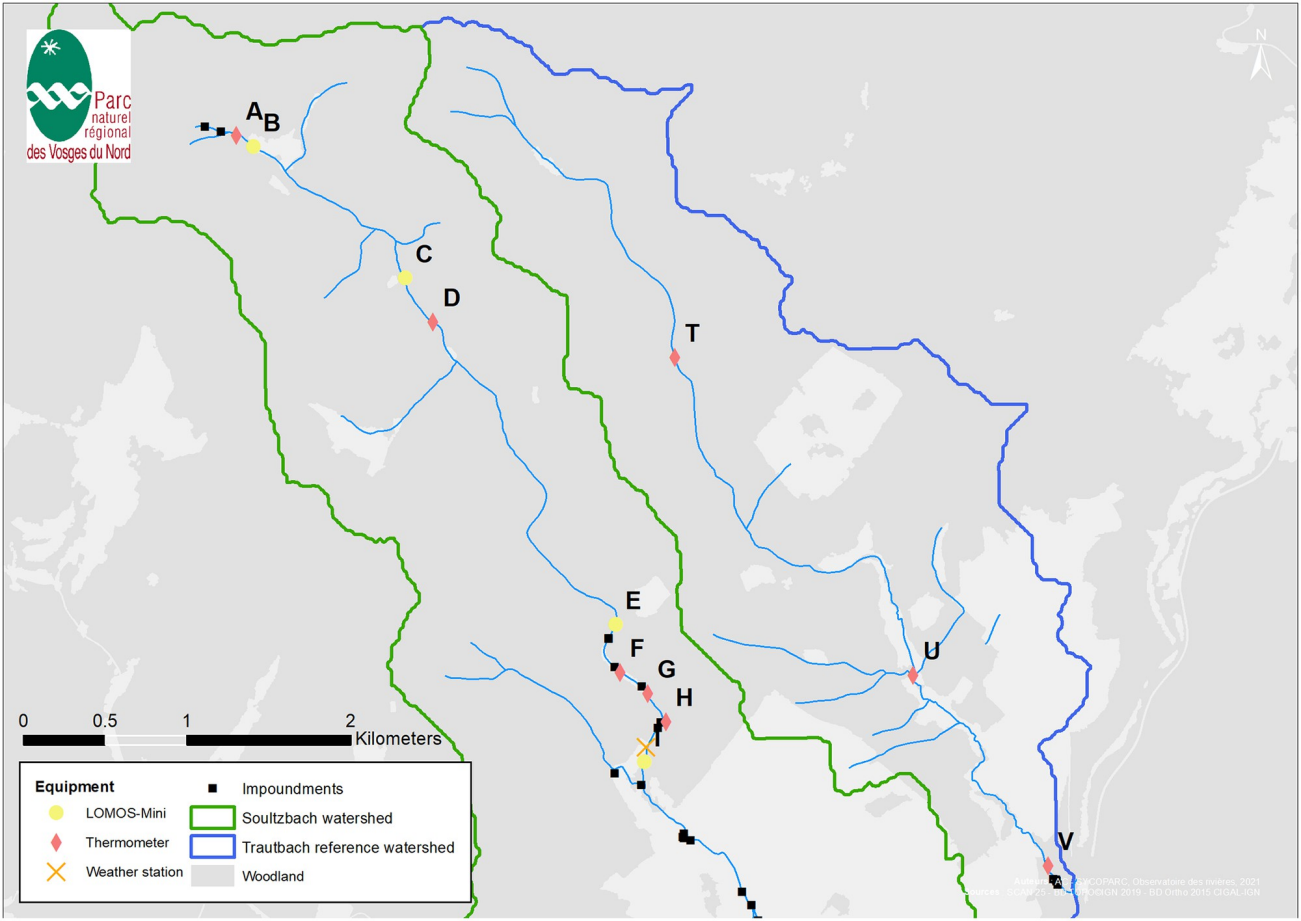
Study streams, watersheds and distribution of sensors on the field along the Soultzbach (West watershed) and the Trautbach (East). All tributaries in the study area are ephemeral and were not active during field study. Impoundments are labeled by their order of stream appearance (Table 1); they are located upstream A, between A and B, between E and F, between H and I.

This study was carried out in the northeast of France (49.0134°N. 7.6931°E), near the France-Germany border. The climate is semi-oceanic, with average daily temperatures ranging between 0°C and 27°C for the study period (S1A Fig). Precipitation in the area ranges between 900 and 1000mm cumulated average rain per year (1971–1990 interannual mean precipitation, with 110–140 days of rain and 20 to 40 of snow) [15], 674 mm for the study period (S1B Fig). Two streams, the Soultzbach (11 km long for the whole stream, 6.5 km for the study reach) and the Trautbach (7 km long), have been monitored during this study. These streams are located at the very head of two adjacent small, forested watersheds (19.9 km^2^ for the whole stream but 9.4 km^2^ for the study reach and 9 km^2^, respectively), similarly oriented in a low-mountain landscape (the Northern Vosges). They both flow through grassland and forest from West to East between +315m and +169m altitude in the Soultzbach, respectively +270m and +180m in the Trautbach (Fig 1). Trautbach stream is only disturbed by forest management and pasture, which makes it a reference for our study; additional disturbances on Soultzbach stream will be detailed below. Apart from this, the two streams share the same geographical orientation (North-West upstream to South-East downstream) and geological features (S1C Fig).

The surrounding forests are principally made of beech, man-introduced spruce, pine, oak and ash and represent roughly 97% of the land cover on the Soultzbach watershed, 70% for the Trautbach watershed. The riparian vegetation types range from open grassland to semi-natural deciduous and conifer woodlands. The geological underground is contrasted with a profile change along the course of the streams (Charm database, BRGM. S1C Fig). Soultzbach typical discharge values are around 2 l/s at the stream source and 40 l/s at the lowest part of the stream.

Four localized disturbances along the Soultzbach result from water impoundments located 0 km (labelled #1), 0.2 km (labelled #2), 4.7 km (labelled #3) and 5.6 km (labelled #4) downstream from the source respectively (Table 1, Fig 1). They display the following features (by order of appearance along the stream course):

1. diffuse runoff water is channelized into a unique stream source, flowing from the impoundment;
2. run-of-the-river impoundment, widening of the streambed, followed by a weir;
3. same as #2;
4. made of two close by-pass impoundments arranged in cascades and fed by a fraction diverted from the stream main flow. This fraction flows back into the stream after the impoundment that was built as fishing ponds for the local fishing club.

**Table 1:** Impoundment features.

	Distance from the source (m)	Maximum water depth (m)	Impoundment length (m)	Impoundment surface (m^2^)	Impoundment volume (m^3^)	Connection type
**#1**	0	0.68	30	560	400 (estimated)	Run-of-the-river
**#2**	200	1.34	78	1,800	1,560 (estimated)	Run-of-the-river
**#3**	4,700	2.36	178	5,240	10,636 (estimated)	Run-of-the-river
**#4**	5,600	1.45	304 (by-pass)	230+2,370	3,847 (estimated)	By-pass

The main local human activities before the nearest village (Langensoultzbach, located 6.4 km downstream from the source) are thus forestry in the surrounding woods and fishing in impoundments #2 and #3. There is only one house within the Soultzbach watershed, and it is a secondary home; all things considered, the watershed remains quite empty of people except for lumberjacks, fishermen and trail-runners. On the contrary, wild game abounds in the area, which can be a source of degradation, e.g., when wild boars cross the stream and pick up thermometers in their wake. Field data collection.

Field monitoring was designed to get stream and hyporheos temperature, flow direction between stream and hyporheos, and climatic parameters (Fig 1). From the complete monitoring period, data from June 11^th^ to December 11^th^, 2019 were selected (completeness of all data series). During this monitoring period, tributaries of the streams, who are all ephemeral, were not active. We also observed that the streambed was homogeneous and did not observe any clay on the streambed; thus, chances that the streambed was clogged were very reduced. Finally, we posit that this streambed homogeneity creates homogeneous thermal transfer conditions across the stream.

Stream temperature was monitored with Hobo U22 thermometers (Onset, Bourne, Massachusetts, USA. Accuracy: ±0.21°C from 0° to 50°C) distributed along the stream to investigate overall evolution and potential singular locations (grassland or forest landscape, presence of impoundments, substrate evolution) (Fig 1, Table 2, S1C Fig). The impact of impoundments on water temperature was specifically monitored by placing thermometers directly up- and downstream of each: #1 surveyed downstream (A), #2 surveyed up- (A) and downstream (B), #3 surveyed up- (F) and downstream (G), #4 discharge surveyed up- (H) and downstream (I) (Fig 1). An hourly time step was used to capture diel dynamics. Data collection on the field was done regularly to try and compensate for potential sensor loss. Thermometers were firmly attached onto low points in the streambed to avoid emerged thermometers in low flow conditions.

**Table 2:** Location of temperature monitoring stations on each stream.

Stream	Soultzbach									Trautbach		
**Station label**	A	B	C	D	E	F	G	H	I	T	U	V
**KP (m)**	0	245	1,870	2,240	4,710	5,000	5,140	5,560	5,620	10	2,350	4,250

The kilometric point (KP) indicates distance from the stream source. Green background indicates forest landscape, while orange background indicates open grassland landscape. For the Soultzbach, the double vertical line indicates the presence of an impoundment.

Hyporheos temperature and pressure were monitored with LOMOS-Mini sensors [16] using calibration data for each sensor to determine pressure gradient between surface water and groundwater with a 2mm precision on piezometric head gradient. Briefly, they are coupled pressure and temperature sensors that allow determining: i) head difference between stream and aquifer and ii) temperature profile in the streambed. These devices allow us to determine the hydrogeological input in the stream heat budget. Streambed temperatures are used to identify losing and gaining reaches by considering heat as a water flow tracer [17]. Groundwater temperature is generally cooler than stream temperature, whereas in winter it is generally the opposite. Additionally, the diurnal stream temperature fluctuates, while the groundwater temperature is constant over a day. This differing behavior allows us to discriminate the orientation of the potential water exchanges between the stream and the aquifer, by calculating the time lag between stream and hyporheos temperature series. They were distributed at four locations along the stream (B, C, E and I) to capture various configurations: B, C, E and I are located on different geological substrates observed on the field (cf. S1C Fig). Measurements were taken every 15 minutes. Data were collected once in July and once in December, as the sensor is water sensitive and needs to be open to retrieve data. Climatic parameters were monitored with an RX3000 weather station (Onset, Bourne, Massachusetts, USA) located next to the stream. Hourly measurements were made to capture diel dynamics. Briefly, pressure data collected with these sensors show that in the upstream part of the Soultzbach surface water mostly feeds the hyporheos (87% of the time at site B and 85% of the time at site C), while downstream the stream is mostly fed by groundwater (100% of the time at site E and 91% of the time at site I).

### Data analysis

All data analyses were performed with R software [18]. Significance of inter-site differences were assessed following the method of [8], as serial autocorrelation of all-time series was first established (Durbin-Watson tests, examination of autocorrelation/partial autocorrelation plots [19]). A mixed-effects model (lme4 package, [20]) was used to determine whether temperature differences were significant (*p*-value: 0.05). Similarity groups were determined (emmeans package [21]). For LOMOS-Mini, time lag between series is computed as the time at which the maximum of cross-correlation function between time series is reached [22]. Results of statistical tests are presented in S1B Table.

## Results

### Stream temperature dynamics

Overall, mean stream temperatures were significantly different across streams and sites (p-value < 0.05), except for A-D, G-I and E-T-U (long forest stretches) points (Table 3, S1B Table). Surface water temperature increased along the studied stretch of the Soultzbach (+2.2°C, equivalent to +0.4°C/km), but this increase concealed contrasting evolution (Table 3). Indeed, leaps of surface means daily water temperature were observed between A and B (+3.7°C), H and I (+1.8°C) and E and F (+1.7°C). Each of these increases followed the singularities on the stream stretch: run-of-the-river impoundments (between A and B, E and F) or outflow from the by-pass impoundment between H and I. Thus, across the study period, the warmest daily averaged temperatures were obtained after the impoundments, at quite close dates (S1A Table). The temperature increased between A and B faded away until D, as daily mean temperature remained stable between C and D, after an average 3.6°C drop between B and C, equivalent to -2.2°C/km. A 0.4°C warming occurred then across the 2.5 km forest stretches between D and E. Downstream F site, a 0.2°C cooling was observed through the 140 m long open grassland (equivalent to -1.4°C/km), and a 1.7°C cooling was observed through the 420 m long following forest stretch (equivalent to -4.0°C/km).

**Table 3:** Daily mean, minimum, maximum, and standard deviation of stream temperature.

			*Whole period*				*Summer*				*Fall*				
		KP (m)	mean	Min	Max	SD	mean	Min	Max	SD	mean	Min	Max	SD	
*Soultzbach*	Source impoundment														
	A	0	11.8	4.2	24.4	3.2	14.1	9.9	24.4	1.6	8.8	4.2	12.6	2.2	
	Run-of-the-river impoundment														
	B	245	15.5	7.0	24.8	3.8	18.2	12.3	24.8	2.0	12.0	7.0	15.9	2.5	
	C	1,870	11.9	3.4	22.7	3.0	14.0	9.0	22.7	1.3	9.3	3.4	13.1	2.3	
	D	2,240	11.8	3.4	19.6	2.8	13.7	8.9	19.6	1.1	9.4	3.4	13.3	2.3	
	E	4,710	12.4	0.6	20.7	4.1	15.2	8.5	20.7	1.8	8.8	0.6	13.7	3.2	
	Run-of-the-river impoundment														
	F	5,000	14.1	3.4	21.2	5.1	18.0	13.5	21.2	1.8	9.3	3.4	13.8	3.5	
	G	5,140	13.9	2.9	21.8	5.2	17.8	13.1	21.8	1.8	9.0	2.9	14.0	3.6	
	H	5,560	12.2	4.2	18.3	3.1	14.4	10.3	18.3	1.3	9.5	4.2	13.3	2.5	
	Outflow from by-pass impoundment														
	I	5,620	14.0	4.2	21.5	4.3	17.3	12.6	21.5	1.6	9.9	4.2	14.0	2.9	
*Trautbach*	T	10	12.5	0.7	21.0	4.2	15.4	9.3	21.0	1.9	8.8	0.7	13.8	3.4	
	U	2,350	12.4	2.3	20.1	3.2	14.6	9.0	20.1	1.3	9.7	2.3	14.6	2.8	
	V	4,250	13.1	3.1	20.7	3.8	15.7	9.9	20.7	1.7	9.7	3.1	14.0	2.9	

Summer: from June 11^th^ to September 20^th^. Fall: from September 21^st^ to December 11^th^. Green background indicates forest landscape, while orange background indicates open grassland landscape. The kilometric point (KP) indicates distance from the stream source. Dates when minimal and maximal temperatures were reached are indicated in S1A Table.

**Fig 2:**
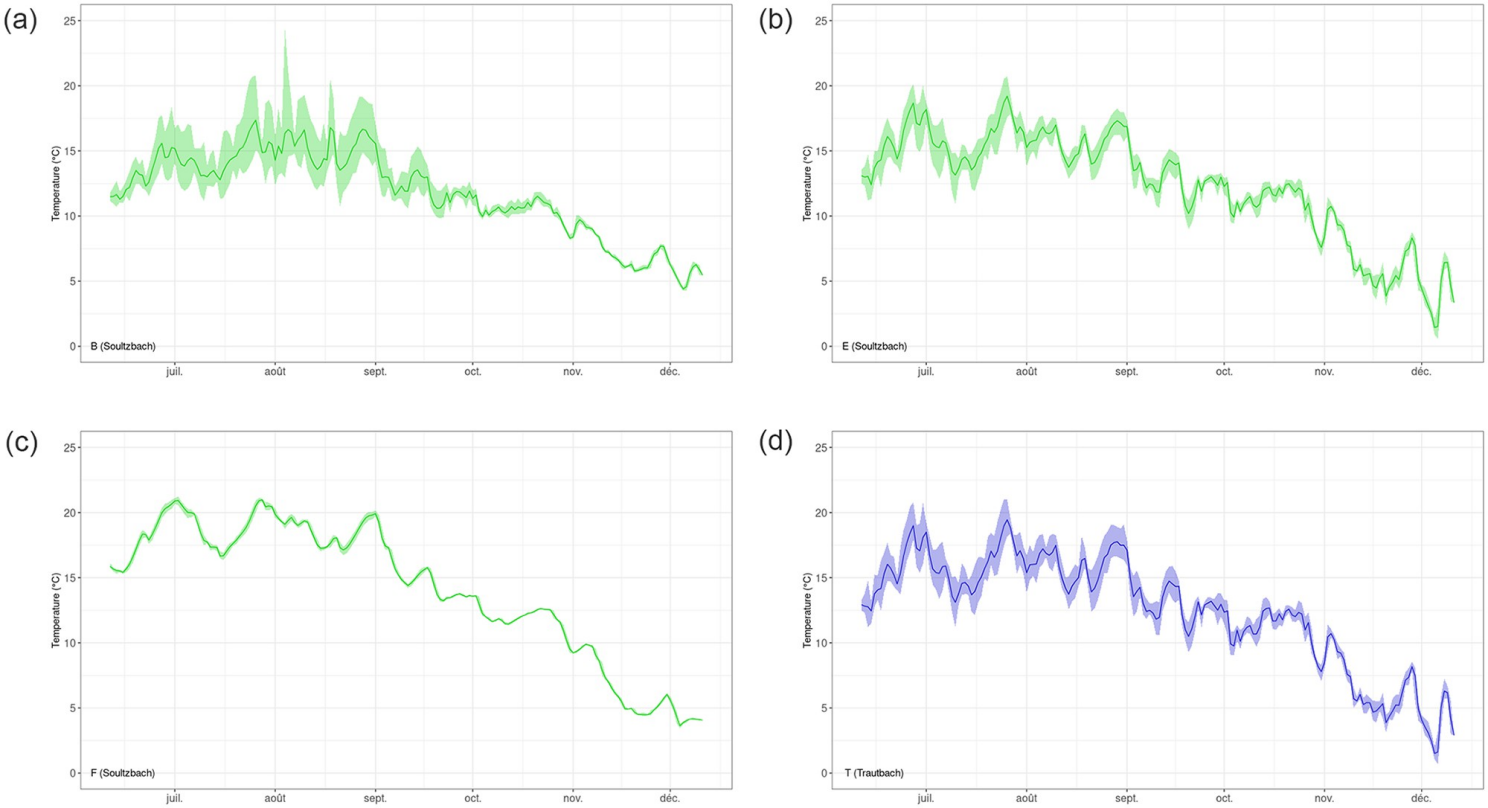
Surface water temperature time series during the study. Green: Soultzbach stream. Blue: Trautbach stream. Letters indicate monitoring stations (see Fig 1).

For the reference stream (Trautbach), water temperature at the source was significantly higher than for the impacted stream (+0.7°C) but warming along the reference stretch was almost 3 times smaller (+0.6°C, equivalent to +0.14°C/km) than along the impacted one. The same seasonal pattern was observed at all measurement points (Fig 2).

During summer, stream temperature was significantly different across streams and sites (*p*-value < 0.05) (Table 3, S1B Table). Highest temperatures (24.8°C, respectively 24.4°C) were reached after the first impoundment, respectively after the source run-of-the-river impoundment.

During fall, temperatures levelled towards baseline values, as temperatures between A, E and T, between U and V, between C and E, and between D and H points were not significantly different.

**Fig 3:**
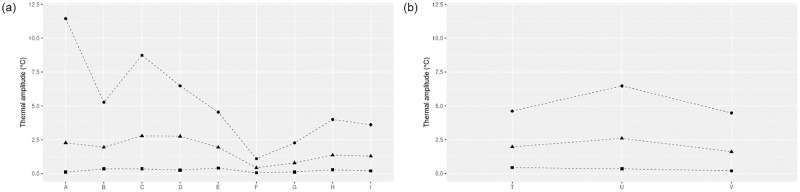
Thermal amplitude (daily maximum temperature—daily minimum temperature) along Soultzbach (a) and Trautbach (b) streams. Letters indicate monitoring stations. Red line: maximum thermal amplitude across the study period. Green line: minimum thermal amplitude across the study period. Blue line: mean thermal amplitude across the study period.

**Fig 4:**
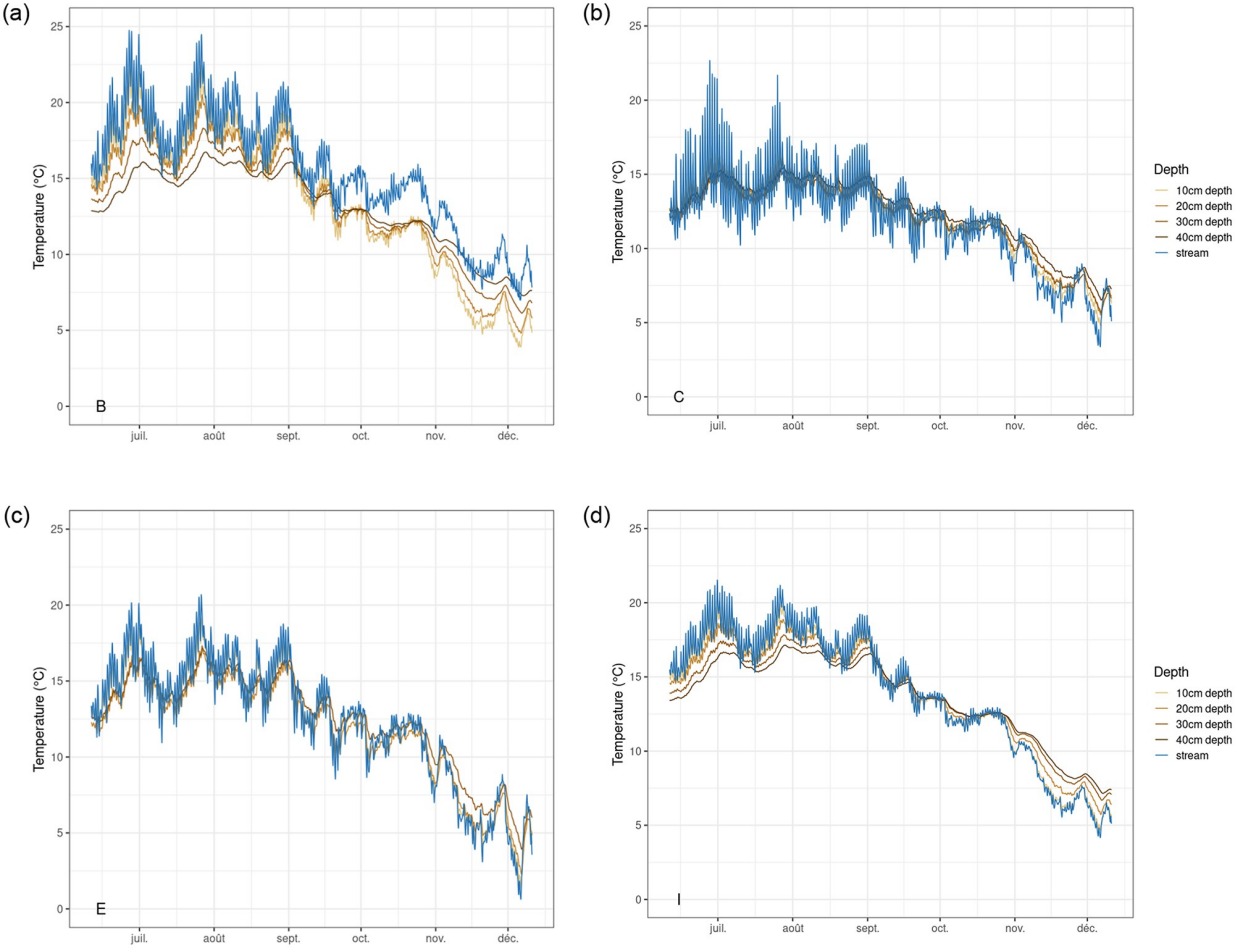
Temperature time series in the stream and adjacent hyporheos. Upper-left quadrant: B station. Upper-right quadrant: C station. Lower-left quadrant: E station. Lower-right quadrant: I station. Blue line: stream temperature. Brown lines: hyporheos temperature at increasing depths, from 10cm-deep (pale brown) to 20cm-, 30cm- and 40cm-deep (dark brown).

When looking at the thermal amplitude for each stream (Fig 3), diurnal temperature amplitude decreased after the first run-of-the-river impoundment (Fig 4a, point B) from 11.4°C to 5.3°C maximal amplitude, and from 2.3°C to 1.9°C mean amplitude. It did accordingly after the second run-of-the-river impoundment (Fig 4a, point F), from 4.5°C to 1.1°C maximal amplitude, and from 1.9°C to 0.4°C mean amplitude. High maximum amplitude was observed at point A, decreased at point B and increased again at point C (Fig 3a). Another local maximum amplitude was observed on point H. Compared to these observations, temperature amplitudes from the Trautbach remained lower, in the range of 0.2°C-6.5°C for all locations and metrics (Fig 3b). On this reference stream, the highest maximum and mean amplitudes were reached after the grassland configurations. Diurnal temperature variability was higher in summer (Fig 2); it receded during fall, for all sites except the ones impacted by impoundments (Fig 2, stations B, E and T, and unpublished data). Yet the decrease of diurnal temperature amplitude was observed all year long, as can be seen on the temporal plot after the second run-of-the-river impoundment (Fig 2, station F); thermal variability observed before the impoundment (Fig 2, point E) was flattened right after the impoundment (Fig 2, point F) and did not recover the pre-impoundment values, as can be seen at station G (S1D Fig), located 150m downstream of the impoundment.

### Hyporheos temperature dynamics

First, all the hyporheos temperature time series fluctuated with the daily mean temperature of the river, while thermal variability was attenuated in the hyporheos (Fig 4). We observed contrasting behaviors along the stream, though: at B and I site, attenuation of diurnal dynamics was observed around 40 cm depth during summer and around 30 cm depth during fall. At C and E sites, diurnal surface temperature dynamics effect was observed in the hyporheos temperature time series during summer, while the signal was almost flattened around 40 cm depth for C, 30 cm depth for E during the fall. Additionally, here as well contrasting depth-related temperature patterns were observed between E and I sites: attenuation of the stream temperature was more important for I site than for E site. B and I on one hand, C and E on the other hand, displayed similar depth-related temperature patterns in terms of thermal variability.

For all points, increasing lag time went along with increasing depth (Table 4): from 2 to 3h at 10 cm depth for all points (equivalent to 12–18 min/cm) to more than half a day at 30 cm (equivalent to 17 to 25 min/cm). The maximum observed lag was 18.5h at 40 cm depth for B site, which corresponded to more than half a day delay.

**Table 4:** Lag time (in h) between surface water and hyporheos maximum temperature at different depths.

*Depth*	B	C	E	I
*10cm*	3	3.5	2	2
*20cm*	6.5	6.5	5	6
*30cm*	12.5	8.5	8.5	11.5
*40cm*	18.5	10.5	Nd	14.5

Positive values indicate delay compared to the surface water. B, C, E and I are the measurement sites. Nd: not determined (sensor failure).

**Fig 5:**
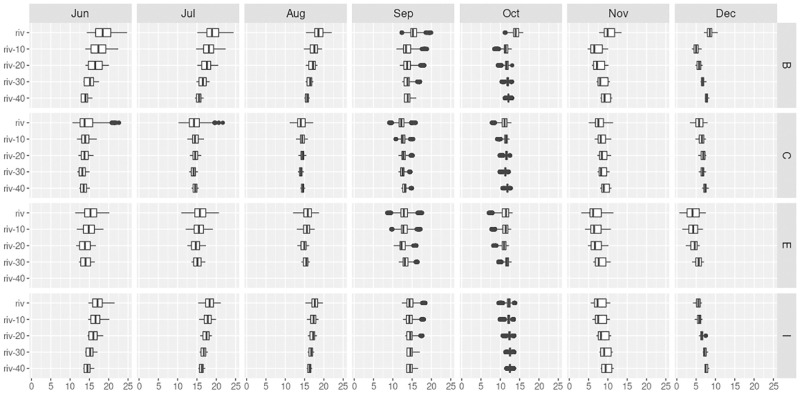
Boxplot of mean hourly temperature along the seasons (from June to December), along the stream (from B to I site) and with depth (blue: Surface water temperature; shades of brown: Hyporheos water temperature).

Seasonal effect on spatial dynamics could be observed (Fig 5). First, there was a temperature shift towards colder values as the season moved from summer to autumn. Moreover, we could see a shift in temperature distribution: from warmer at the surface and cooler in the hyporheos in June for all sites, to cooler at the surface and warmer in the hyporheos in December for all sites as well. The transition between these two distinct distribution patterns occurred between September and October, when marked outlier temperature values were recorded and during which the depth-related temperature ***gradient*** almost nullified. Temperature outliers were otherwise not found at other periods, except for surface water at C site during summer. At monitoring station B, there was a singular behavior at 10 cm below the stream, as it was cooler than both surface and deeper horizons from October to December.

To describe more quantitatively depth-related temperature gradient, we chose to focus on values observed in sites C and I (Table 5), as they showed contrasted main water fluxes direction (mainly downward for C, upward for I; see “Data Collection”) and landscapes (C is surrounded by grassland, I by forest). Similar data layout for sites B and E are shown in S1C Table. A consistent warming was observed from June to December at site C between the surface and 20 cm below, as the mean temperature difference between surface and 10cm-depth hyporheos ranged between -0.16°C in June down to -0.74°C in December and reaches -0.33°C at maximum between 10 and 20 cm below the surface, reaching a 0.18°C warming in June. For site I, cooling with depth occurred for the whole sampled zone from June to August; the trend reversed around September-October, to establish then, as warming with depth was observed for the whole sampled zone in November and December.

**Table 5:** Mean monthly temperature difference with depth.

		Jun	Jul	Aug	Sep	Oct	Nov	Dec
**C**	**T**_**riv**_ **—T**_**riv-10**_	-0.16	-0.26	-0.36	-0.35	-0.38	-0.62	-0.74
	**T**_**riv-10**_ **—T**_**riv-20**_	0.18	-0.06	-0.12	-0.23	-0.13	-0.28	-0.33
	**T**_**riv-20**_ **—T**_**riv-30**_	0.56	0.42	0.39	0.22	0.30	0.20	0.12
	**T**_**riv-30**_ **—T**_**riv-40**_	-0.36	-0.42	-0.47	-0.58	-0.53	-0.69	-0.62
**I**	**T**_**riv**_ **—T**_**riv-10**_	0.52	0.47	0.31	0.05	0.05	-0.16	-0.24
	**T**_**riv-10**_ **—T**_**riv-20**_	0.57	0.34	0.13	-0.25	-0.24	-0.72	-0.69
	**T**_**riv-20**_ **—T**_**riv-30**_	0.81	0.69	0.46	-0.05	-0.17	-0.82	-0.63
	**T**_**riv-30**_ **—T**_**riv-40**_	0.71	0.52	0.42	0.12	0.03	-0.40	-0.28

The top row indicates months of the year; letters in the left column indicate site location. Blue cells indicate cooling with increasing depth, red cells indicate warming with increasing depth. Brighter cells, respectively paler cells indicate higher, respectively lower magnitude.

## Discussion

### Stream temperature dynamics

Overall, impoundments and landscapes significantly influenced stream thermal regime at a local scale: impoundments created up to +3.7°C temperature increase in average (Table 3), and generated visible amplitude dampening (Fig 2), while open grassland landscapes generate higher thermal amplitude than forest ones (Fig 2). The effect of varying hydrogeological features on stream thermal regime could not be identified, though. The mean increase rate of Soultzbach stream surface temperature was +0.4°C/km, the same order of magnitude as previously monitored streams (+0.6°C/km in [2]). During fall, radiation decrease causes surface temperatures to level towards baseline values, as temperatures became not significantly different (p-value 0.05) across the Soultzbach stream (between A/E/T, C/F, and D/H respectively), as well as across the Trautbach stream (U/V).

The observed warming on the Soultzbach was three times higher than the one observed on the reference stream (+0.14°C/km on the Trautbach stream) (Table 3). And indeed, each of the three impoundments leads to an increase of temperature, consistent with their overflow discharge mode, with observed values in the range of previous studies [23–26]. In particular, similar temperature increases were reported in [25], with similar discharges. The warming effect of the first impoundment drops then between the impoundment outlet and the following monitoring station, 1.6km downstream (corresponding to -2.2°C/km). This recovery of thermal balance (+3.6°C in average) falls in the range of previous results on streams with similar Strahler order [24, 25, 27, 28]. This may be due to the low discharges of the stream and a subsequent reduced thermal inertia, conjugated with colder water side supply by affluent groundwater (see the ‘Hyporheos temperature section’ for further discussion), that counteracts the large share of solar radiation received by the stream on the grassland reach. To end up on the effect of impoundments on stream temperature, temperature increase is logically smaller in the case of the by-pass impoundment, as only a fraction of the stream flow is diverted into it.

Diurnal stream temperature variability is higher in summer, when the sun trajectory is higher above the tree line; it recedes during fall, for all sites (stations in Fig 2 and unpublished data) except downstream of the second run-of-the-river impoundment (F). Yet the dampening effect of the impoundment is acting through the whole year, as can be seen on the temperature time series at the same location. The dampening effect created by impoundments causes maximal and mean thermal amplitude to drop after the first and second run-of-the-river ponds (Fig 3). This pond-dampening effect was reported in [4, 28, 29], although the effect is less marked (< 1.3°C amplitude decrease) in [29], probably due to the size of the streams (stream order 3–5, while only 1 in this study). In the case of our study stream, a minimum thermal amplitude recovery distance might be estimated, as thermal variability is not recovered 150m downstream (G, S1D Fig) of the impoundment discharge (F) that created a strong dampening effect. A surprisingly high maximum amplitude at what is considered as the stream source (A), may be explained by the presence of the impoundment (#1) that collects source water before discharging it as a stream. The small volume of the impoundment, and its exposition to solar radiation make it prone to warming during the summer months, while the volume of the downstream ponds makes them more prone to thermal stratification and amplitude dampening (Fig 3a, 3B and 3F). Eventually, another local maximum amplitude is observed in the second forest section (H), maybe due to reduced thermal inertia of the stream, as a significant fraction of the flow is diverted towards the by-pass impoundment.

The effect of grassland and forest on stream temperature appears on both temperature itself and thermal amplitude. The same seasonal pattern of stream temperature at all measurement points corresponds to radiation main influence (Fig 2), as reported in [2, 3, 7, 13]. Downstream of the first run-of-the-river impoundment though, a moderate warming occurs across the 2.5 km forest stretch (equivalent to +0.16°C/km); as radiation is reduced along the forest landscape, this highlights the significant effect of radiation in the thermal regime of this stream. On another variable, the grassland landscape probably allows solar radiation to fully influence stream thermal amplitude as seen amidst the grassland section (C). The high maximum amplitude of temperature observed on the latter section was shown in several publications, among which [13, 30]. By comparison, preserved stream temperature amplitudes (Fig 3b) remain modest across the forest landscape for all locations and metrics, with an increase of temperature amplitude downstream the grassland area.

### Hyporheos temperature dynamics

Overall, the influence of landscape and management (i.e., impoundments) features on hyporheos thermal regime is not shown here, while there is quite logically a stronger effect of hydrogeological features. Indeed, hyporheos thermal regime varies from stream regime by temperature dynamics delay (up to 18h) and decrease (up to -7°C between surface and hyporheos temperature in average).

Temperature buffering observed in the hyporheos (Fig 4) depends on the water exchanges between the stream and the aquifer through the streambed [31–37]. Streambed exchanges qualitatively differ between sites, with various buffering across them. These differences along the Soultzbach stream are probably due to contrasting hydrodynamic and thermal properties of the encountered substrates [37]. More advanced modeling with an appropriate model [16, 38] (DOI: 10.5281/zenodo.4058821) needs to be carried out to decipher these complex thermal exchanges. Logically, differences of geological features (S1C Fig) and landscape variability are intertwined to produce the observed temperature patterns observed during monitoring with LOMOS sensors.

The pattern of diurnal delay (Table 4), from a few hours at shallow depth to more than half a day at the highest depth, advocates for variably efficient thermal exchanges occurring within the hyporheos. This is probably partly due to variable streambed composition: silica sand in the upstream and colluvium in the downstream (field observation). Delays similar to the ones measured in our study were observed in a sandstone bed stream with much greater discharge [36]. This consistent increasing delay with depth shows another feature of the hyporheic zone: the shift of peak temperature at a daily scale. Thus, thermal shelter effect comes not only from amplitude dampening but also from time lag from a few hours to more than half a day between the different depths (Table 4), which corresponds to an anti-correlation at the day scale for some spots.

A shift in temperature distribution between seasons was observed for all sites (Fig 5): from warmer at the surface and cooler in the hyporheos in June, to cooler at the surface and warmer in the hyporheos in December. A transition period was recorded between September and October, when strong outlier temperature values were recorded, as seen in [36]. Temperature outliers are not found at other periods, except for surface water in the grassland section (C) site during summer, when solar radiation hits the river most across the grassland landscape. The impact of contrasting landscape (grassland versus forest) on temperature-depth gradient most probably conjugates with geological features to produce the observed patterns; yet further study is required to quantify the contribution of each exchange process. Still, it is in agreement with previous studies showing the existence of spatially- and temporally dynamic gradients in the hyporheic zone [10], such as in the study of [39] where an unexpectedly high thermal heterogeneity of hyporheic water was found, examining the thermal regime of surface and hyporheic waters within the channel network of a glacial floodplain for 1 year. In the study stream, right after the first run-of-the-river impoundment (B), a singular behavior was found just below the stream: cooler than both surface and deeper horizons, from October to December. This heterogeneity may be due to the potential colder water supply by affluent groundwater, as already reported in Table 3 for mean temperature.

A consistent yet moderate warming is observed between the surface and 20 cm depth from June to December in the grassland section (C, Table 5); the mean temperature difference between surface and 10cm-depth hyporheos ranged between -0.16°C in June down to -0.74°C in December and reached -0.33°C at maximum between 10 and 20 cm below the surface, reaching however a 0.18°C cooling in June. For the most downstream monitoring station (I), cooling with depth occurred across the whole bed from June to August; the trend reversed around September-October towards warming with depth across the whole bed in November and December. This illustrates the supply of water from the aquifer, cooler than surface water in summer, and warmer than surface water in winter.

The effect of hydraulic fluxes seems entangled with geological features, as contrasting depth-related temperature patterns were observed for the first two monitoring sites (B and C), where the stream flowed into the hyporheos. Yet the effect of stream temperature fluctuation in the hyporheic zone was buffered on the two last monitoring sites (E and I); the aquifer flowed into the stream, so temperature variations were buffered by the constant aquifer temperature advected by the upward flux. Comparing the two forest monitoring sites (E and I), attenuation of the stream temperature was more important for the most downstream one (I), which might be due to greater aquifer-water fluxes at this location. Contrasting landscape was not the only significant driver of the hyporheos thermal regime, as both grassland and forest depth-related temperature patterns were similar in terms of thermal variability (B and I, respectively C and E). No significant effect on hyporheos thermal patterns was found, though. This result, contrasting from the ones reported in [40, 41], could be explained by hydrogeological and climatic differences between these study streams and ours.

## Conclusion

An increase of mean temperature was observed along the impacted stream, with contrasting magnitude and amplitude caused by the combined effect of impoundments and landscape variations. Contrasting landscapes showed significant mean temperature decreases, and seasonal effects were marked for every measurement site. Along the stream, diurnal thermal amplitudes are strongly reduced in the hyporheic zone, although seasonal variations remain significant. Temporal patterns are delayed up to more than 12h with increasing depth in the hyporheos. Cooling or warming patterns are strongly influenced by geological features of the streambed, yet in our study no impoundment effect was observed in the hyporheos. Eventually, we found that contrasting climatic, landscape, anthropic and pedological factors generate a mosaic of thermal habitats across a longitudinal-vertical gradient along the stream. Due to thermal dampening in the hyporheic zone, some points in this zone remain as cool as at the stream source and thus could be shelter zones for temperature-sensitive species if morphological criteria allow it. From an analytical perspective, the investigation of hyporheos thermal regime can be carried out in very diverse ways, from highly technological and resource intensive manner [42] to the low-cost material used in this study [38]. Low-cost material, through further development aiming at a more user-friendly set-up, could allow operational stakeholders to understand and use these devices on the field to study restoration works at reduced resource cost.

To expand on an ecological perspective, headwater stream ecosystems are sensitive to climate change [43]; in our case, thermal disturbance expected from global change is in the range of the impact of a unique run-of-the-river pond. Indeed, significant changes in biological traits may result from small temperature shifts [44]. Moreover, various alterations due to climate change (e.g., temperature, hydrology and atmospheric composition) disturb multiple levels of biological organization, also in freshwater ecosystems [8]. Thus because of the ecological importance of stream temperature, and in this context, preventing, mitigating or restoring anthropogenic thermal degradation has become a common concern for resource managers. A run-of-the-river pond appears clearly a double jeopardy for the ecological status of headwater stream: it significantly alters the thermal regime of surface water (shown in this study, as well as in others) and hyporheic water [40, 41], as a result (among others) of a low thermal inertia due to small discharges. Thermal heterogeneity of the habitat may be reduced and lead to cancelling the thermal shelter function for freshwater fauna. Thermal disturbances also influence functional processes (e.g., leaf litter decomposition) at the basis of trophic chains in forested headwater streams, generating additional disturbances. A way to escape from this disturbance could be to migrate towards more favorable habitats [8], but habitats fragmentation by impoundments also modify flow and disturb natural dispersal of aquatic organisms. Another way, more anthropically directed, would be to erase such impoundments to recover a less disturbed landscape. Ongoing works on the study site will achieve this, and joint physical and ecological effects of this modification should be assessed in the near future.

Following this first study, further steps will be necessary to determine the energy balance of the stream. Thermal habitats and their ecological counterparts could be subsequently superimposed to characterize biological communities on sandstone headwater stream in surface and hyporheos water, and further study the ecological effect of thermal habitats on stream and hyporheos biocenosis.

## Supporting information

S1 Fig (DOCX)

S1 Table (DOCX)

S1 File (PDF)
