# Supplementary material for: Water temperature dynamics in a headwater forest stream: Contrasting climatic, anthropic and geological conditions create thermal mosaic of aquatic habitats
Source: PLoS One. 2023 Feb 15;18(2):e0281096. doi: 10.1371/journal.pone.0281096 (PMC9931118; doi:10.1371/journal.pone.0281096)

Supplementary information

Figure A: Air temperature during monitoring period


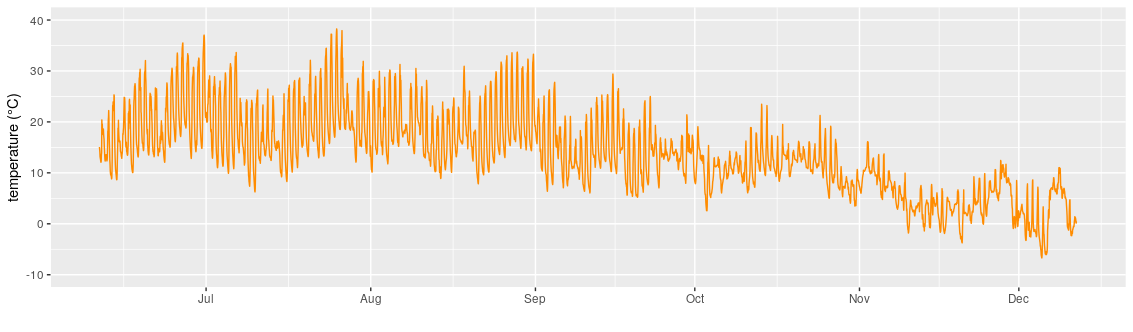


Figure B: Rainfall during monitoring period


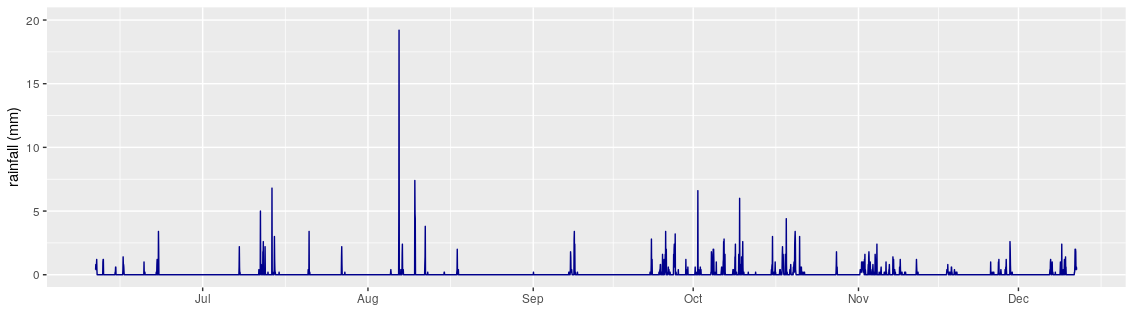


Figure C: Geological map of the study site. Watersheds are outlined in green (Soultzbach) and blue (Trautbach). Sensors are located for comparison purpose with Figures 3 & 4. Republished from SYCOPARC under a CC BY license, with permission from Alban Cairault, original copyright 2019.


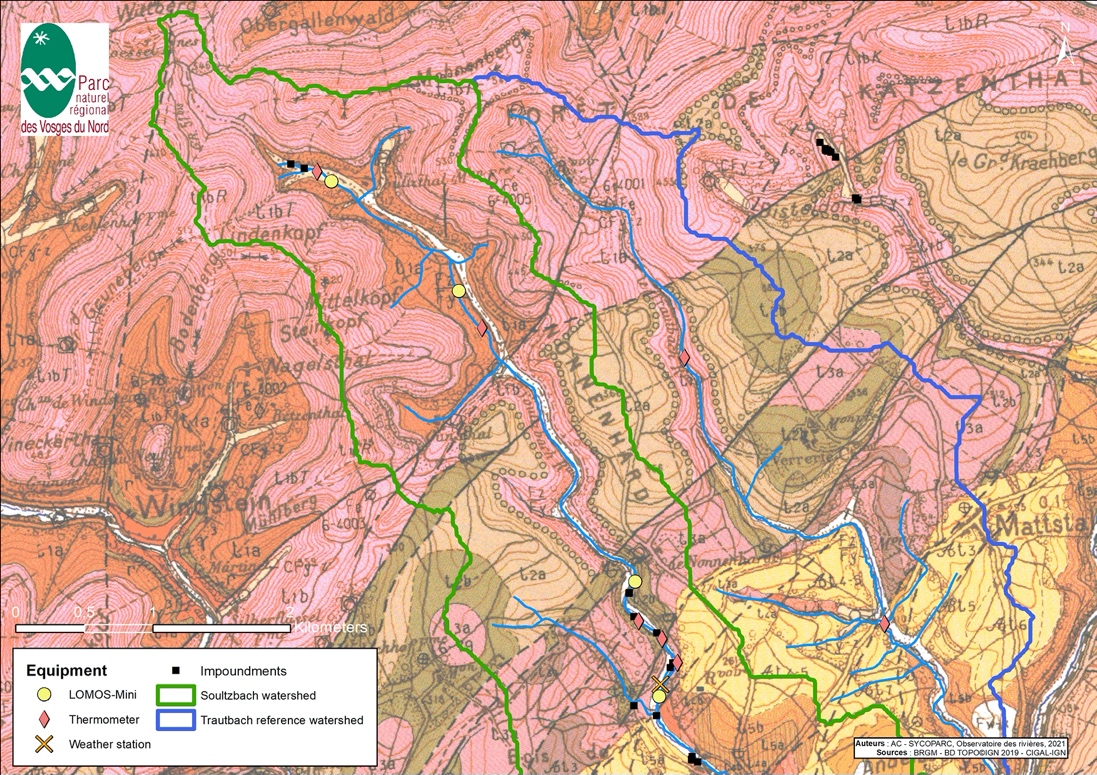


Figure D: Surface water temperature time series during the study at monitoring site G on Soultzbach stream


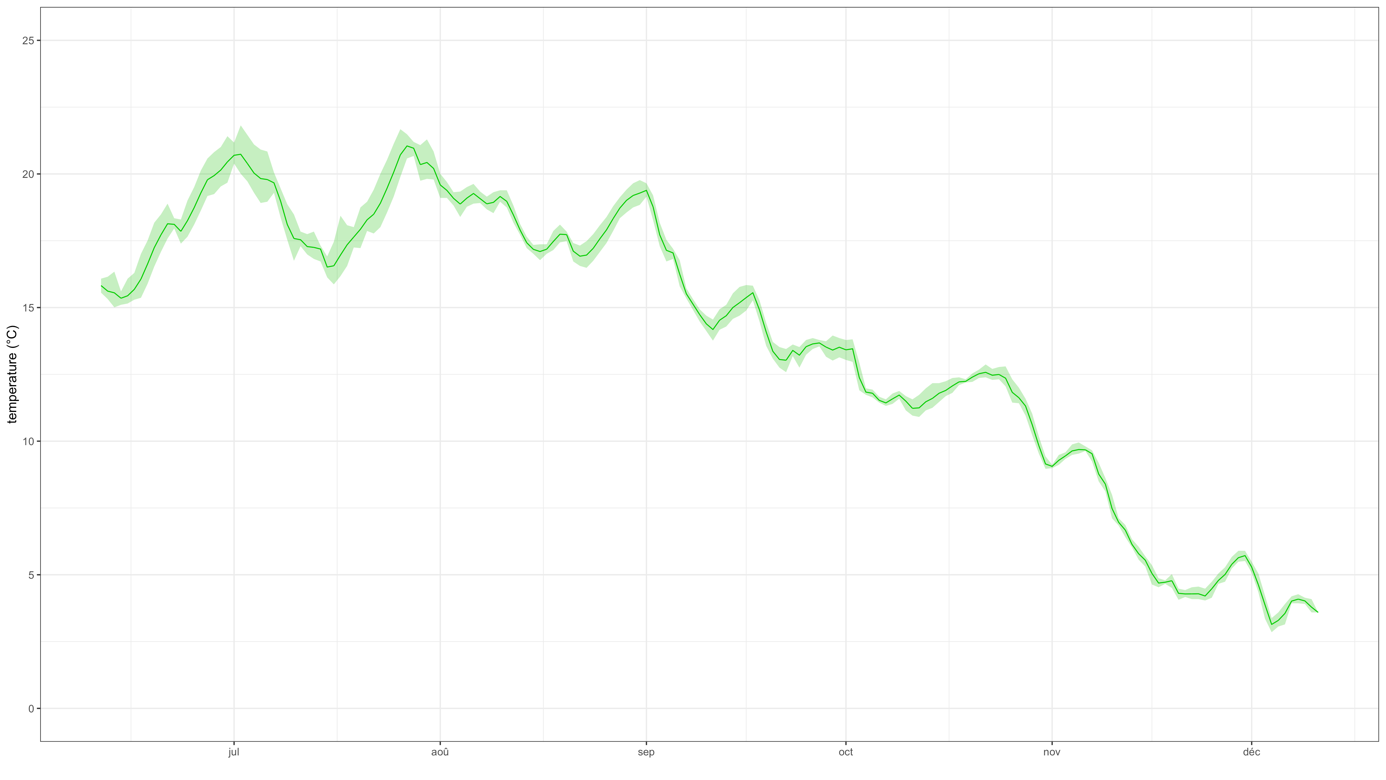

Supplement: S1 Fig — (DOCX) [file pone.0281096.s001.docx]
