# Supplementary material for: Water temperature dynamics in a headwater forest stream: Contrasting climatic, anthropic and geological conditions create thermal mosaic of aquatic habitats
Source: PLoS One. 2023 Feb 15;18(2):e0281096. doi: 10.1371/journal.pone.0281096 (PMC9931118; doi:10.1371/journal.pone.0281096)
Supplement: S1 Table — (DOCX) [file pone.0281096.s002.docx]

Table A: Date of extreme mean temperatures (minimum and maximum). Green background indicates forest landscape, while orange background indicates open grassland landscape. The kilometric point (KP) indicates distance from the stream source.

|  |  | **KP (m)** | **Min (°C)** | **Date of Min** | **Max (°C)** | **Date of Max** |
| --- | --- | --- | --- | --- | --- | --- |
| Soultzbach | A | 0 | 4.2 | 12/06/2019 | 24.4 | 08/04/2019 |
|  | B | 245 | 7.0 | 12/06/2019 | 24.8 | 06/26/2019 |
|  | C | 1,870 | 3.4 | 12/06/2019 | 22.7 | 06/27/2019 |
|  | E | 2,240 | 3.4 | 12/06/2019 | 19.6 | 06/27/2019 |
|  | F | 4,710 | 0.6 | 12/06/2019 | 20.7 | 06/26/2021 |
|  | H | 5,000 | 3.4 | 12/04/2019 | 21.2 | 07/02/2021 |
|  | I | 5,140 | 2.9 | 12/04/2019 | 21.8 | 07/02/2019 |
|  | J | 5,560 | 4.2 | 12/05/2021 | 18.3 | 06/27/2019 |
|  | L | 5,620 | 4.2 | 12/06/2019 | 21.5 | 06/30/2019 |
| Trautbach | T | 10 | 0.7 | 12/06/2019 | 21.0 | 07/25/2019 |
|  | U | 2,350 | 2.3 | 12/06/2019 | 20.1 | 08/28/2019 |
|  | V | 4,250 | 3.1 | 12/06/2019 | 20.7 | 07/26/2019 |

Table B: p-values for statistical comparison tests. Corresponding tests were performed on temperature time-series (cf. Table 3 and ‘Results section’), following the methodology described in the ‘Material and Methods’ part (see ‘Data analysis’ section). Sites range from A to L (Soultzbach) and T to V (Trautbach). Bold figures correspond to non-significant differences (threshold value: 0.05). All other p-values not reported in this table were < 10^-4^.

| **Whole period** | A | C | D | E | G | I | T | U |
| --- | --- | --- | --- | --- | --- | --- | --- | --- |
| A |  |  |  |  |  |  |  |  |
| C | < 10^-4^ |  |  |  |  |  |  |  |
| D | **0.9981** | 0.0027 |  |  |  |  |  |  |
| E | < 10^-4^ | < 10^-4^ | < 10^-4^ |  |  |  |  |  |
| G | < 10^-4^ | < 10^-4^ | < 10^-4^ | < 10^-4^ |  |  |  |  |
| I | < 10^-4^ | < 10^-4^ | < 10^-4^ | < 10^-4^ | **0.8551** |  |  |  |
| T | < 10^-4^ | < 10^-4^ | < 10^-4^ | **0.8181** | < 10^-4^ | < 10^-4^ |  |  |
| U | < 10^-4^ | < 10^-4^ | < 10^-4^ | **1.0000** | < 10^-4^ | < 10^-4^ | **0.9945** |  |

| **Fall** | A | C | D | E | H | T | U | V |
| --- | --- | --- | --- | --- | --- | --- | --- | --- |
| A |  |  |  |  |  |  |  |  |
| C | < 10^-4^ |  |  |  |  |  |  |  |
| D | < 10^-4^ | 0.0165 |  |  |  |  |  |  |
| E | **1.0000** | **0.9390** | < 10^-4^ |  |  |  |  |  |
| H | < 10^-4^ | < 10^-4^ | **0.0923** | < 10^-4^ |  |  |  |  |
| T | **0.7398** | < 10^-4^ | < 10^-4^ | < 10^-4^ | < 10^-4^ |  |  |  |
| U | < 10^-4^ | < 10^-4^ | < 10^-4^ | < 10^-4^ | < 10^-4^ | < 10^-4^ |  |  |
| V | < 10^-4^ | < 10^-4^ | < 10^-4^ | < 10^-4^ | < 10^-4^ | < 10^-4^ | **0.0820** |  |

Table C: Mean monthly temperature difference with depth. Letters in the left column indicate site location. Blue cells indicate cooling with increasing depth, red cells indicate warming with increasing depth. Brighter cells, resp. paler cells indicate higher, resp. lower magnitude.

|  |  | **Jun** | **Jul** | **Aug** | **Sep** | **Oct** | **Nov** | **Dec** |
| --- | --- | --- | --- | --- | --- | --- | --- | --- |
| **B** | **T_riv_ - T_riv-10_** | 1.11 | 0.80 | 1.05 | 1.79 | 2.59 | 3.46 | 3.54 |
|  | **T_riv-10_ - T_riv-20_** | 0.84 | 0.51 | 0.36 | -0.29 | -0.21 | -0.70 | -0.76 |
|  | **T_riv-20_ - T_riv-30_** | 1.34 | 1.05 | 0.72 | -0.09 | -0.25 | -0.83 | -0.97 |
|  | **T_riv-30_ - T_riv-40_** | 1.18 | 1.00 | 0.74 | -0.09 | -0.24 | -1.14 | -0.88 |
| **F** | **T_riv_ - T_riv-10_** | 0.45 | 0.26 | 0.22 | 0.14 | 0.06 | -0.25 | -0.19 |
|  | **T_riv-10_ - T_riv-20_** | 0.99 | 0.79 | 0.68 | 0.48 | 0.45 | -0.22 | -0.40 |
|  | **T_riv-20_ - T_riv-30_** | -0.16 | -0.41 | -0.49 | -0.88 | -0.77 | -0.99 | -1.04 |

Table D: Time differences (in days) for given degree-day values considering water temperature of hyporheos (at different depth) vs. surface water temperature. B and C indicate sensor location. For each location, time difference is calculated with the surface point as a reference. Cumulative DD at the end of monitoring can be compared with reference DD for surface water at F and L: resp. 2,269, 2,581. Positive values indicate later reach of it.

| **DD** | **F** | | | | **L** | | | |
| --- | --- | --- | --- | --- | --- | --- | --- | --- |
|  | ***10cm*** | ***20cm*** | ***30cm*** | ***40cm*** | ***10cm*** | ***20cm*** | ***30cm*** | ***40cm*** |
| **200** | 1 | 2 | 1 | - | 0 | 1 | 1 | 2 |
| **400** | 1 | 3 | 2 | - | 1 | 2 | 3 | 4 |
| **600** | 1 | 3 | 2 | - | 1 | 3 | 4 | 6 |
| **800** | 1 | 4 | 3 | - | 2 | 3 | 5 | 7 |
| **1,000** | 1 | 5 | 4 | - | 2 | 3 | 6 | 8 |
| **1,200** | 2 | 6 | 4 | - | 2 | 4 | 7 | 10 |
| **1,400** | 2 | 7 | 4 | - | 2 | 4 | 7 | 10 |
| **1,600** | 2 | 8 | 3 | - | 3 | 5 | 9 | 12 |
| **1,800** | 2 | 9 | 3 | - | 3 | 5 | 9 | 13 |
| **2,000** | 3 | 12 | 3 | - | 4 | 6 | 10 | 14 |
| **Cumulative DD** | 2,254 | 2,173 | 2,295 | - | 2,541 | 2,548 | 2,525 | 2,494 |
