# Supplementary material for: Water temperature dynamics in a headwater forest stream: Contrasting climatic, anthropic and geological conditions create thermal mosaic of aquatic habitats
Source: PLoS One. 2023 Feb 15;18(2):e0281096. doi: 10.1371/journal.pone.0281096 (PMC9931118; doi:10.1371/journal.pone.0281096)
Supplement: S1 File — (PDF) [file pone.0281096.s003.pdf]

Request for Permission to Publish Content under CC BY 4.0 License

Dear Rights Holder or Representative,

I have submitted a paper for publication in a journal by PLOS, the Public Library of Science (https://plos.org/). I am hereby requesting permission to use content under your or your organization's management within this paper.

PLOS journals are published under a Creative Commons Attribution 4.0 International license (CC BY 4.0), which allows for unrestricted distribution as well as non-commercial and commercial use of all PLOS journal articles and content, as long as attribution is given to the creator or rights holder of the content. See the full CC BY 4.0 license terms here: https://creativecommons.org/licenses/by/4.0/legalcode.

By granting permission to use this content within a PLOS article, you agree to allow the content's publication under a CC BY 4.0 license without restriction.

To grant me permission to use the content in my PLOS paper, please enter your name, today's date, and your signature on the second page of this form and return the completed form to me at my email address. You may either use an electronic signature or print, sign, and scan or photograph the form.

Thank you very much for your consideration of this request.

Author Name:

Alban Cairault

Author Email Address:

albancairault@hotmail.fr

Description of the content which I am seeking permission to use:

Two maps presenting the study site

Link to the Content:

* * *

On behalf of myself or the rights holder, I hereby grant the permission sought herein.

Copyright Holder Name and Title:

Alban Cairault, chargé de mission rivière (SYCOPARC)

Date:

8/31/22

Signature of Party Granting Permission:

6bc77c75-8426-487c
-8648-a8baba6c12d0

Signé numériquement par
6bc77c75-8426-487c-8648-a8baba6c12d0
ND : CN=6bc77c75-8426-487c-8648-a8baba6c12d0
Raison : Je suis l'auteur du document
Emplacement : l'emplacement de votre signature ici
Date : 31-08-2022 16:05:14
Foxit Reader Version: 10.0.1
